# Signs and Symptoms of Vitamin D Deficiency in Children: A Cross-Sectional Study in a Tertiary Pediatric Hospital in the United Arab Emirates

**DOI:** 10.7759/cureus.18998

**Published:** 2021-10-23

**Authors:** Fatma Alawadhi, Lemis Yavuz

**Affiliations:** 1 Pediatrics, Al Jalila Children's Hospital – Mohammed Bin Rashid University of Medicine and Health Sciences (MBRU), Dubai, ARE

**Keywords:** vitamin d deficiency, vitamin d level, public awareness of vitamin d, pediatrics, prevalence rate

## Abstract

Objective: This study was conducted to estimate the common signs and symptoms present in patients with vitamin D deficiency at a children’s specialty hospital in Dubai, United Arab Emirates (UAE).

Methods: This descriptive cross-sectional study (n = 805) examined patients aged <18 years with a serum 25-hydroxy vitamin D concentration of <50 ng/mL and presenting to the hospital between 2017 and 2018. Variables (age, gender, nationality, vitamin D levels, signs and symptoms, and primary complaint) were described using frequencies and mean values (SD). Chi-square and Kruskal-Wallis tests were conducted.

Results: Among the 805 patients, 315 (39.2%) had vitamin D deficiency (<20 ng/mL). Regarding the symptoms of these 315 patients, 26% (n = 82) of them were asymptomatic, 13.3% (n = 42) of them had endocrine symptoms and other/rare symptoms, and 11.7% (n = 37) of them had gastrointestinal symptoms. The least common symptoms were found in the mixed category (mixed symptoms of different body systems), consisting of 3.5% (n = 11) of patients. Vitamin D deficiency was more common among female patients (44.8%) and Emiratis (40.5%), and the average age for patients to have vitamin D deficiency was nine years.

Conclusion: To our knowledge, this is one of the first studies in the United Arab Emirates to focus on and examine patients with low vitamin D levels in detail. Determining the most frequent symptoms is helpful for healthcare practitioners because our results showed that most patients with the deficiency were asymptomatic. Hence, we recommend performing regular checkups for healthy and asymptomatic children to detect vitamin D deficiency before they show any symptoms.

## Introduction

Vitamin D is one of the most important vitamins in our body. It is produced from 7-dehydrocholesterol, which later produces pre-vitamin D3 with the help of ultraviolet B radiations. Vitamin D3 then binds to the serum vitamin D-binding protein and travels through the circulation to the liver, where it is metabolized and converted into 25-hydroxyvitamin D (25(OH)D). 25(OH)D is converted into its final form 1,25-hydroxyvitamin D3 in the kidney [1,2]. Recent evidence implicates the essential involvement of vitamin D metabolites in a host of cellular processes, including calcium homeostasis, immunity, cell differentiation, and gene transcription regulation [3]. Vitamin D deficiency is diagnosed through a blood test for the level of 25-hydroxyvitamin D in the blood. Most studies have defined 25-hydroxyvitamin D levels of <20 ng/mL as vitamin D deficiency [4]. Based on this cutoff level, the prevalence of vitamin D deficiency was approximately ≥70% in South Asia, and it varied from 6% to 70% in Southeast Asia [5]. In most clinical scenarios, vitamin D deficiency is asymptomatic and underdiagnosed. However, in severe cases, patients may present with daytime fatigue [6], increasing pain [7,8], and bone problems [9,10], and novel studies are starting to link vitamin D deficiency with autism [11,12]. Most of these symptoms can be avoided by providing the patient with vitamin D supplements [13].

Unfortunately, in the United Arab Emirates (UAE), no sufficient accurate data describe this common condition in children, due to which there is limited information available to the general population. This situation necessitates the significance of research on vitamin D deficiency; hence, data collection and analysis in this regard could help several medical practitioners in the UAE to increase their awareness concerning the importance of vitamin D.

Therefore, the aims and objectives of the present study were to (a) analyze and determine the symptoms and signs present in patients diagnosed with vitamin D deficiency in Al Jalila Children’s Hospital (AJCH) in 2017 and 2018, (b) calculate the frequency of symptoms and determine the common symptoms, and (c) compare the symptoms and the level of vitamin D between the other variables such as gender, nationality, and age. This investigation is a descriptive cross-sectional study; the hypothesis will be generated at the end of the study and can be later proven through analytical research.

## Materials and methods

The following information was formulated according to the Strengthening the Reporting of Observational Studies in Epidemiology guidelines:

Ethics

Data were collected anonymously, and ethical approval was obtained from Mohammed Bin Rashid University of Medicine and Health Sciences (MBRU) (approval number MBRU - IRB - 2019).

Study design and setting

This descriptive, cross-sectional study was conducted to analyze the signs and symptoms of children diagnosed with vitamin D deficiency at Al Jalila Children’s Hospital (AJCH) in Dubai, UAE. Data previously recorded from January 2017 to December 2018 in an electronic database in the AJCH record system were retrieved in March 2019.

Participants

The study participants were children aged <18 years and diagnosed with vitamin D deficiency and insufficiency and those who required supplements and follow-up. The diagnosis was confirmed through a blood test to check for 25-hydroxyvitamin D concentration in the blood. The inclusion criteria were children aged <18 years with a vitamin D level of <50 ng/mL and presenting to AJCH in 2017 and 2018. The exclusion criteria were children with complex diseases that can cause vitamin D deficiency, such as patients with chronic gastrointestinal tract diseases, patients with eating disorders, and patients with diseases with multisystem involvement.

Variables

The patients were divided into different subgroups based on the level of vitamin D, age, nationality, signs and symptoms, primary complaint, and gender. The study variables were divided into categorical and continuous data. Vitamin D level, signs and symptoms, primary complaint, gender, and nationality were the categorical variables, whereas age was the only continuous variable used in this study.

Data measurements and quantitative variables

As mentioned earlier, the collected data were divided into categorical and continuous variables. Age was the only continuous variable as it is measured in years. Gender and nationality each have two groups. For the variables symptoms, signs, and primary complaint, we divided all the symptoms that we recorded into eight categories: 1, asymptomatic/checkup; 2, orthopedic; 3, cardiopulmonary; 4, gastrointestinal; 5, endocrine; 6, general (common symptoms that are nonspecific, such as fatigue and headaches); 7, others (rare symptoms or psychological symptoms); and 8, mixed (symptoms from mixed body systems). Vitamin D level was measured on a numeric scale and then divided into three groups based on the hospital’s guidelines (Table 1) [13].

**Table 1:** Vitamin D status of the study participants as per the National Osteoporosis Foundation cutoffs [13]

Category	Level
1: Deficiency	<20 ng/mL
2: Insufficiency	21–29 ng/mL
3: Supplements and monitoring	30–50 ng/mL

Study size

The sample size used in this study depended on a time frame that commenced from the start of 2017 and concluded at the end of 2018. Therefore, the sample size included any child who presented to the hospital and was diagnosed with this condition within the specified time frame and met the eligibility criteria. The sample size used in this study consisted of 805 children.

Statistical analysis

Data were exported from the laboratory in AJCH as a Microsoft Excel spreadsheet, and analyses were conducted using the Statistical Package for Social Sciences, version 24. Descriptive statistics were used to describe the characteristics of the variables using frequencies for categorical variables or measures of tendency and dispersion for the continuous variable. Differences between the subgroups of the sample (age, gender, vitamin D level, primary complaint, nationality, and signs and symptoms) were analyzed using a chi-square test for categorical variables and ANOVA for the continuous variable, which are normal. Normality was evaluated using the Shapiro-Wilk test. If continuous variables, such as age, were not normal, the Kruskal-Wallis test was used. The first objective was to calculate the frequency of the signs and symptoms and the primary complaint in patients with vitamin D deficiency only (<20 ng/mL). The second objective was to compare vitamin D levels and signs and symptoms with other variables using the previously mentioned tests. The results are represented as either tables or diagrams (bar and line graphs). An alpha value ≤ 0.05 and a p value < 0.01 were applied to determine statistical significance.

## Results

**Figure 1:**
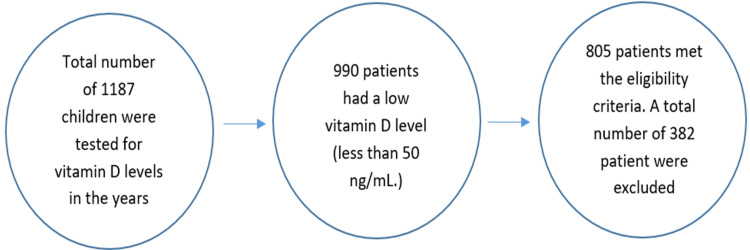
Patient population

A total of 1187 patients were tested for vitamin D levels in the years 2017 and 2018, of which only 805 patients met the eligibility criteria to be included in this study (Figure 1).

Among those 805 patients, 94.2% were Emiratis (n = 758) and 5.8% were non-Emiratis (n = 47), and 52% were male (n = 419) and 48% were female (n = 386). The majority (39.1%) of patients had vitamin D deficiency; i.e., they had a 25-hydroxyvitamin D level of <20 ng/mL (n = 315). In the majority of patients (33.3%), the primary complaint was discovered upon a general checkup (n = 268), which implies that the patient presented to the hospital with no initial specific or significant complaints. Of the patients, 26.7% were asymptomatic (n = 215), which is generally expected among patients visiting for a general checkup, considering that the frequency of symptoms and the primary complaint are for the entire study group and not specific to patients with vitamin D deficiency (Table 2).

**Table 2:** Demographic characteristics, vitamin D levels, primary complaint, and signs and symptoms of the participants

Variable	Number of participants
Nationality	
Emirati	758 (94.2%)
Non-Emirati	47 (5.8%)
Gender	
Female	386 (48%)
Male	419 (52%)
Vitamin D level	
Deficiency	315 (39.1%)
Insufficiency	313 (38.9%)
Supplements and monitoring	177 (22%)
Primary complaint	
Checkup	268 (33.3%)
Orthopedic	82 (10.2%)
Cardiopulmonary	111 (13.8%)
Gastrointestinal	82 (10.2%)
Endocrine	84 (10.4%)
General	65 (8.1%)
Others	107 (13.3%)
Mixed	6 (0.7%)
Signs and symptoms	
Asymptomatic	215 (26.7%)
Orthopedic	87 (10.8%)
Cardiopulmonary and gastrointestinal	108 (13.4%) and 84 (10.4%)
Endocrine	82 (10.2%)
General	93 (11.6%)
Others	109 (13.5%)
Mixed	27 (3.4%)

The mean age of the patients was 7.39 (SD = 4.124) years (Table 3).

**Table 3:** Mean age and standard deviation

Age	
Number	
Valid	805
Missing	0
Mean	7.39
Standard deviation	4.124

Of the 805 patients who met the eligibility criteria, 382 were excluded. A total of 990 patients had a low vitamin D level (<50 ng/mL). All the 1187 children were tested for vitamin D levels in the years 2017 and 2018.

**Figure 2:**
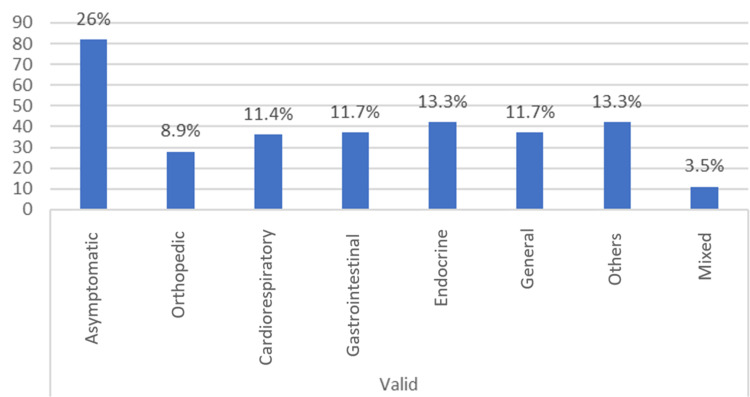
Frequency of signs and symptoms

**Figure 3:**
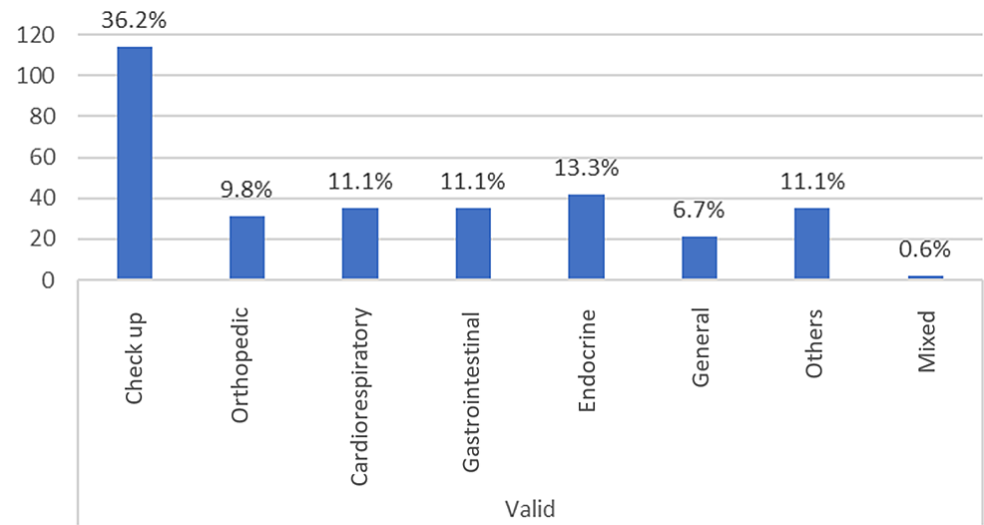
Frequency of primary complaint

Frequency data were tabulated, and a diagram was plotted using the data of the 315 patients who had vitamin D deficiency. Most patients in this study group (26%) presented as asymptomatic with vitamin D deficiency (n = 82). The majority of them (36.2%) visited the clinic for a general checkup (n = 114). The least common symptoms and signs were in the mixed category, comprising only 3.5% (n = 11) of patients. A similar result was found for the primary complaint, which comprised only 0.6% in the mixed category (n = 2) (Table 4) (Figures 2, 3). (The percentages were within vitamin D deficiency and not within symptoms and the primary complaint.)

**Table 4:** Percentage within vitamin D deficiency

Variable	Number of participants and percentages
Signs and symptoms	
Asymptomatic	82 (26%)
Orthopedic	28 (8.9%)
Cardiopulmonary	36 (11.4%)
Gastrointestinal	37 (11.7%)
Endocrine	42 (13.3%)
General	37 (11.7%)
Others	42 (13.3%)
Mixed	11 (3.5%)
Primary complaint	
Checkup	114 (36.2%)
Orthopedic	31 (9.8%)
Cardiopulmonary	35 (11.1%)
Gastrointestinal	35 (11.1%)
Endocrine	42 (13.3%)
General	21 (6.7%)
Others	35 (11.1%)
Mixed	2 (0.6%)

When the association between vitamin D levels and other variables was evaluated, a statistical significance was found (p < 0.05) between vitamin D levels and gender, as well as nationality and age. A majority of female patients (44.8%; n = 173) had vitamin D deficiency, and a major proportion of male patients (42.7%; n = 179) had vitamin D insufficiency. In terms of nationality, 40.5% of Emirati patients (n = 307) had vitamin D deficiency, and 51.1% of non-Emirati patients (n = 24) were more likely to have vitamin D insufficiency. In this study group, the average age of patients with vitamin D deficiency was 9.18 (SD = 3.794) years, and that of patients with vitamin D insufficiency was 6.89 (SD = 3.89) years. Regarding signs and symptoms, most patients who presented as asymptomatic (40.5%) had vitamin D insufficiency (n = 87), whereas most patients who presented with endocrine symptoms (51.2%) had vitamin D deficiency (n = 42). Regarding the primary complaint, 42.5% of patients who visited for a general checkup were found to be deficient (n = 114). Similar to the result concerning signs and symptoms, 50% of patients with an endocrine problem had vitamin D deficiency (n = 42). (For symptoms/primary complaint, the percentages were within symptoms and the primary complaint and not within vitamin D level.) P value was statistically nonsignificant in both signs and symptoms (p = 0.108) and primary complaint (p = 0.180) (Table 5).

**Table 5:** Association between vitamin D levels and other variables

Variable	Deficiency	Insufficiency	Supplements and monitoring	P value
Gender – n (%)				
Female	173 (44.8%)	134 (34.7%)	79 (20.5%)	0.006
Male	142 (33.9%)	179 (42.7%)	98 (23.4%)	
Nationality – n (%)				
Emirati	307 (40.5%)	289 (38.1%)	162 (21.4%)	0.006
Non-Emirati	8 (17%)	24 (51.1%)	15 (31.9%)	
Age – mean (SD)				
Age	9.18 (3.794)	6.89 (3.89)	5.1 (5.10)	<0.01
Signs and symptoms – n (%)				
Asymptomatic	82 (38.1%)	87 (40.5%)	46 (21.4%)	0.108
Orthopedic	28 (32.2%)	30 (34.5%)	29 (33.3%)	
Cardiorespiratory	36 (33.3%)	40 (37%)	32 (29.6%)	
Gastrointestinal	37 (44%)	34 (40.5%)	13 (15.5%)	
Endocrine	42 (51.2%)	28 (34.1%)	12 (14.6%)	
General	37 (39.8%)	37 (39.8%)	19 (20.4%)	
Others	42 (38.5%)	44 (40.4%)	23 (21.2%)	
Mixed	11 (40.7%)	13 (48.1%)	3 (11.1%)	
Primary complaint – n (%)				
Checkup	114 (42.5%)	104 (38.8%)	50 (18.7%)	0.180
Orthopedic	31 (37.8%)	28 (34.1%)	23 (28%)	
Cardiorespiratory	35 (31.5%)	44 (39.6%)	32 (28.8%)	
Gastrointestinal	35 (42.7%)	31 (37.8%)	16 (19.5%)	
Endocrine	42 (50%)	26 (31%)	16 (19%)	
General	21 (32.3%)	29 (44.6%)	15 (32.1%)	
Others	35 (32.7%)	47 (43.9%)	25 (23.4%)	
Mixed	2 (33.3%)	4 (66.7%)	0 (0%)	

**Figure 4:**
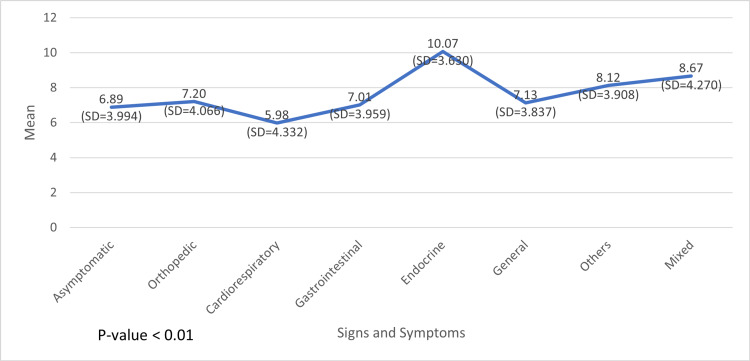
Association between age, and signs and symptoms

When the association between signs and symptoms and other variables was evaluated, statistical significance was found with age, primary complaint, and nationality. The mean age was the highest (10.07 years) in patients who had endocrine-related symptoms (SD = 3.630), and the lowest mean age of 5.98 (SD = 4.332) years was found in patients with cardiorespiratory-related symptoms (Figure 4).

**Figure 5:**
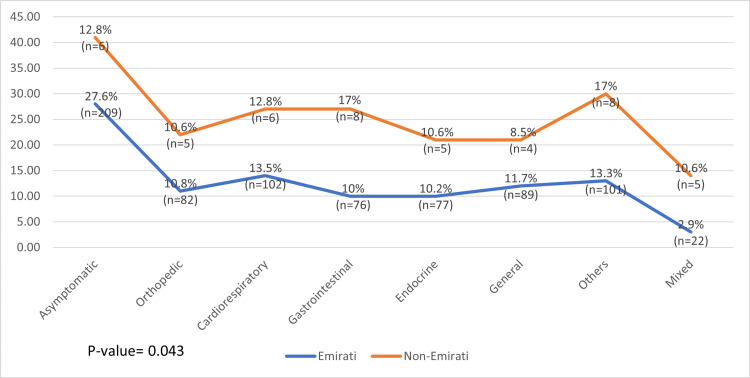
Association between nationality, and signs and symptoms

In terms of nationality, there was a significant difference in signs and symptoms among Emirati and non-Emirati patients, wherein 12.8% of non-Emirati patients were asymptomatic (n = 6) and 17% visited the hospital with other/rare symptoms (n = 8). Among Emirati patients, 27.6% were asymptomatic (n = 209), which was the maximum for these patients. The lowest percentages for both categories were found for mixed symptoms, wherein 10.6% of non-Emirati patients had mixed symptoms (n = 5) compared with 2.9% of Emirati patients (n = 22) (Figure 5).

**Figure 6:**
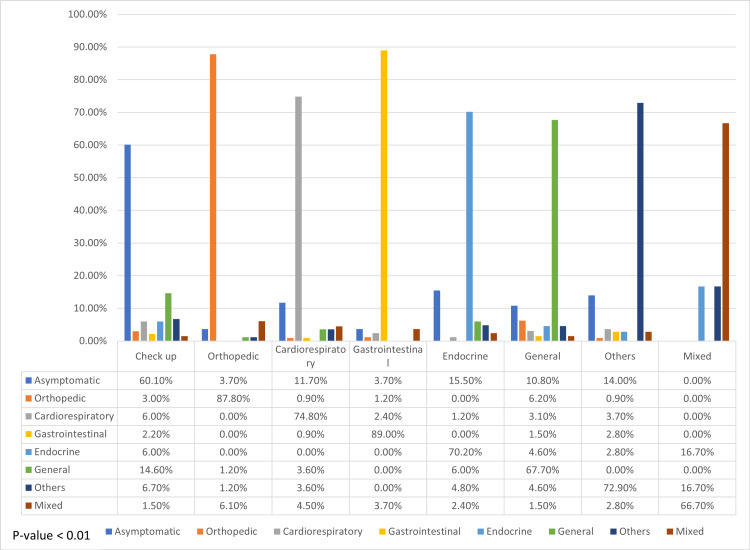
Association between the primary complaint, and signs and symptoms

Another variable that was found to be associated with signs and symptoms was the primary complaint; this result was expected because, at the beginning of the study, patients generally present with symptoms related to their hospital visit. A total of 60.10% of patients who came for a general checkup were asymptomatic (n = 161). However, 87.8% of patients who visited the hospital for an orthopedic problem presented with orthopedic symptoms (n = 72), 74.8% of them who presented with cardiorespiratory symptoms visited the hospital due to a cardiorespiratory problem (n = 83), and 11.7% of patients who had a primary complaint of cardiorespiratory symptoms presented as asymptomatic (n = 13) (Figure 6).

**Figure 7:**
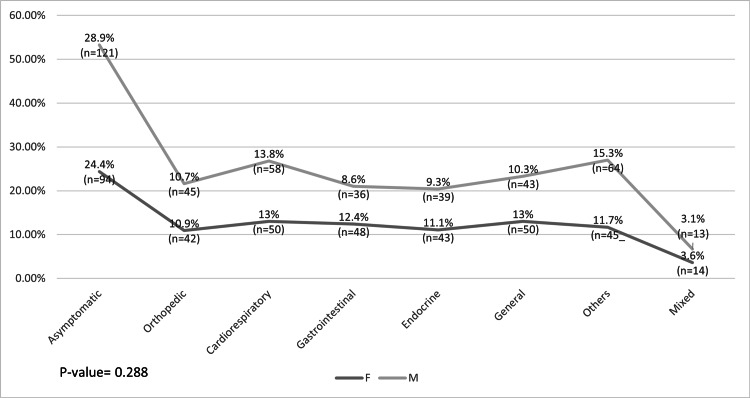
Association between gender, and signs and symptoms

Regarding the association between gender and the symptoms that the patients presented with, 28.9% of male patients presented as asymptomatic (n = 121) compared with 24.4% of female patients (n = 94). The lowest percentages for both were in the mixed symptom category, wherein 3.1% of male patients presented with mixed symptoms (n = 13) compared with 3.6% of female patients (n = 14). The p value was statistically nonsignificant (p > 0.05) (Figure 7).

## Discussion

The primary goal of this study was to investigate the major signs and symptoms of patients with vitamin D deficiency. The study group comprised children aged <18 years who visited a children’s specialty hospital in Dubai, UAE, in 2017 and 2018. Most patients (36.2%) who were diagnosed with vitamin D deficiency visited the hospital for a general checkup. Asymptomatic (26%) presentation was the commonest finding in patients with vitamin D deficiency, and the least common finding was mixed symptoms (3.5%).

Interestingly, we observed that vitamin D levels were associated with gender, nationality, and age. Most patients who had vitamin D deficiency were Emiratis (40.5%) and female subjects (44.8%), and the average age of these patients was 9.18 (SD = 3.794) years. In contrast, we found that patients' symptoms were associated with nationality, age, and primary complaint. As anticipated, most patients who came for a general checkup presented as asymptomatic (60.10%), and regarding the other symptoms, we found them matching with the patients' primary complaint. Patients in the highest mean age category (10 years) mostly presented endocrine-related symptoms, whereas those in the youngest mean age category (5.98 years) had cardiorespiratory-related symptoms. In terms of nationality, a significant difference was found in the symptoms between Emiratis and non-Emiratis, wherein 12.8% of non-Emiratis were asymptomatic compared with 27.8% of asymptomatic Emiratis.

The only study in the literature related to vitamin D deficiency in children in the UAE was the research conducted in Al Ain on 315 adolescents. That study estimated the prevalence of vitamin D deficiency in patients aged 15-18 years. The authors reported that 41 participants (19.7%) had vitamin D deficiency, 143 (45.4%) had vitamin D insufficiency, and an overall 65.1% of the study participants had either vitamin D deficiency or insufficiency [14]. They also found that female patients were more likely to have vitamin D deficiency (28%) than male patients (10%). In our study, we found that 44.8% of female patients had vitamin D deficiency compared with 34.7% of male patients. This supports the claim that gender might be a risk factor for vitamin D deficiency. Another study conducted in Kuwait reported similar results, wherein female subjects were more likely to have vitamin D deficiency than male subjects [15].

Implications for public health practitioners/clinicians/educators

Although there is extensive evidence confirming that vitamin D deficiency is highly prevalent in this region, unfortunately, as mentioned earlier, the information related to this issue is limited for the general public in this region. In the present research, our major goal was to educate the general public through public health educators. Considering the magnitude of the disease burden, it is necessary to initiate a movement of educating schools and parents so that they can adopt preventative methods required to ensure regular and healthy levels of vitamin D in the community. One of the major causes of vitamin D deficiency or insufficiency is the lack of sunlight; hence, informing the parents and the general public about this issue will help decrease the incidence of this disease. One of the major results of our study was that most patients with vitamin D deficiency present as asymptomatic, which implies that public health practitioners should always test for vitamin D levels even if the patient is devoid of any symptomatology related to vitamin D. This will help in the early detection of this condition, after which supplements and monitoring can be used for treatment. It is better to treat the deficiency when there are no symptoms before the patient progresses and becomes symptomatic.

Strengths, limitations, and generalizability

We observed several positive results and significance, which is the major strength of our study. Unfortunately, because of the descriptive cross-sectional study design, the results cannot be generalized to all patients who have vitamin D deficiency. This is due to the study group being recruited from one hospital and during a specific time frame. One of the primary limitations was the lack of references and articles related to this subject, especially in the UAE. This was a major issue because we could not find a background to compare our study data. Coding bias was the major bias that we believe could have influenced our study because we had 805 patients, the symptoms that we found were different from each person, and we had to code all of them into eight groups. This might have influenced our results.

Areas for future research

Vitamin D deficiency is one of the major medical problems affecting the people of this country. However, the general public is still not aware of how this might negatively impact their health, especially children. To date, this is the only study in the UAE to investigate vitamin D deficiency in children. Future studies could focus more on the long-term effect; in fact, a cohort study would be better to follow up patients who had vitamin D deficiency in their childhood and observe how this would affect their adult life. A case-control study could also compare a healthy group and a group of children with vitamin D deficiency. Studies must also be conducted to check whether gender, nationality, and age are risk factors for vitamin D deficiency.

## Conclusions

To our knowledge, this is the first study in the UAE to identify the commonest signs and symptoms that a patient with vitamin D deficiency might exhibit. We also found the variables associated with those symptoms and their correlation with vitamin D levels. This study will be very useful to provide a basic reference for future studies related to this subject. Nevertheless, we recommend conducting more studies so that the general public can be further educated on this issue, especially because vitamin D deficiency is one of the commonest medical problems found in this region. Our primary message for healthcare practitioners is to detect asymptomatic patients with vitamin D deficiency before they exhibit any symptoms.
